# Assessment and management of anorexia nervosa during COVID-19

**DOI:** 10.1017/ipm.2020.60

**Published:** 2020-09

**Authors:** O. Walsh, F. McNicholas

**Affiliations:** 1 CHI at Temple St, Dublin, Ireland; 2 CHI at Crumlin, Dublin 12, Ireland; 3 Lucena Clinic, Rathgar, Dublin 6, Ireland; 4 Department of Child and Adolescent Psychiatry, SMMS, UCD, Dublin 4, Ireland

**Keywords:** Anorexia nervosa, COVID-19, SIMPLE, standardised care

## Abstract

Management of the high rates of medical and psychiatric complications, including self-harm and suicide, associated with anorexia nervosa requires regular clinical review. However, during the current pandemic, face-to-face clinical assessments carry the risk of infection and transmission in this vulnerable cohort already compromised by low weight and lowered immunity. This paper describes how one service has had to adapt usual care during the COVID-19 pandemic without contributing excessively to carer burden or compromising patient safety.

Anorexia nervosa (AN) is the third most common chronic condition after obesity and asthma in adolescent females (Lucas *et al*. 1991; Whitaker, 1992; Golden *et al*. 2003) with prevalence rates for eating ‘problems’ amongst adolescents in Ireland at 10% (McNicholas *et al*. 2010), with 3% reaching clinical thresholds (Merikangas *et al*. 2010; CDC, 2013). AN is characterised by body image distortion and fear of fatness, driving significant calorie restriction, over exercising and at times medically dangerous compensatory behaviours such as post-prandial vomiting, laxative or diuretic use. A sense of lack of personal control has been postulated to play a central role in the aetiology of AN, contributing to treatment dropout (Eivors *et al*. 2003).

The medical complications seen in youth with AN are extensive (see Table 1). Whilst most complications are fully reversible with weight restoration, potentially irreversible damage to growth, bone and reproductive health can occur. High levels of co-morbid anxiety and depression contribute to morbidity and mortality, such that is has the highest mortality risk of all mental health disorders, with deaths related to both suicide and medical complications (Arcelus *et al*. 2011). The increased stress caused by the COVID-19 pandemic and the collective sense of lack of personal control as we abide by government enforced restrictions is likely to affect us all. However individuals with an eating disorder, with mental and physical risks, and their carers, might be more at risk.

**Table 1.** Medical complications in anorexia nervosa

Fluid and electrolytes	Dehydration, electrolyte derangements, oedema
Cardiovascular	Bradycardia, cardiac arrhythmias, orthostatic hypotension
Gastrointestinal	Delayed gastric emptying, constipation, superior mesenteric artery syndrome
Renal	Elevated urea and creatinine, decreased glomerular filtration rate, renal calculi
Endocrine	Primary or secondary amenorrhoea, pubertal delay, growth retardation
Musculoskeletal	Muscle wasting, reduced bone mineral density, increased fracture risk
Dermatologic	Acrocyanosis, lanugo, hair loss
Neurological	Syncope, structural brain changes, decreased concentration

Outpatient treatment is considered the most appropriate setting for the treatment of youth with AN delivered by specialised professionals with emphasis on the role of the family (Hilbert *et al*. 2017). This has been endorsed by the National Clinical Programme for eating disorders (ED) with the development of community-based specialised centres or ‘hubs’. Training in family-based treatment (FBT), accepted internationally as one of the evidence-based approaches to treatment (Lock, 2015), has been rolled out. Careful and skilled medical and psychiatric assessment is an essential component for both the specific treatment planning and delivery. This is especially true in youth, given the high morbidity and mortality associated with AN, and the risk of them becoming medically compromised very rapidly.

Lucena Child and Adolescent Mental Health Services (CAMHS), although not recognised as an ED hub, had recently established a dedicated clinic to assess and treat youth with AN using FBT. Youth referred by GPs with possible eating concerns are initially assessed by multi-disciplinary team (MDT) members from the catchment area generic team, and referred for FBT treatment. Given the multi-disciplinary nature of the FBT team, and the non-standardised assessment or referral information received, there is a risk that salient medical or psychiatry information is not collected at baseline which may lead to clinical risk or treatment delays.

A pilot project called “SIMPLE” (**S**tandardised **I**nclusive **M**edical & **P**sychiatry eva**L**uation of **E**ating Disorders) commenced in January 2020. Using a biopsychosocial assessment model, it proposed a standard framework for the assessment of a number of domains by FBT team members. These included a thorough assessment of (i) individual factors, i.e. eating disorder and co-morbid psychopathology; (ii) family factors including parenting stressors, family history of an eating disorder; (iii) medical assessment and nutritional status; and (iv) environmental factors, including cognitive vulnerabilities, and academic and/or social pressure. A bespoke assessment pro forma was developed, including well-validated questionnaires and cognitive tasks. The FBT team had access to monthly hospital-based inter-professional SIMPLE clinics, with an adolescent medicine paediatrician and dietician with expertise in the management of AN, and the consultant child psychiatrist. Any necessary medical investigations such as blood tests, ECGs and DEXA scans were organised at this clinic. Access to a psychologist to support a neurocognitive battery was also available. It was hoped that by standardising the approach to assessment and management, this might ensure both medical and psychiatry aspects of risk are regularly considered and attention paid to family and environmental factors that may act as barriers to treatment. Seven patients were seen in the first 2 months of 2020.

Clinical services were significantly disrupted following the arrival of a novel coronavirus, SARS-CoV-2 to our shores. SARS-CoV-2 is the aetiological agent of COVID-19 disease which has spread at an alarming pace. Infection rates in youth are generally low accounting for just about 1–2% of reported cases, with few aged under 19 years presenting with severe (2.5%) or critical (0.2%) disease (WHO, 2019; Ludvigsson, 2020).

Compared to adults who typically present with fever, cough and shortness of breath, presentation in youth is more often with non-specific symptoms; rhinitis, pharyngitis, malaise, with or without cough and fever. They are also more likely to have simultaneous infection with another respiratory pathogen, which in many cases account for their respiratory symptoms. More recently, evidence suggests increased risks in individuals who are immunocompromised, and have other medical co-morbidities, both frequent presentations in medically malnourished youth with AN (D’Antiga, 2020).

Whilst AN is not mentioned as a co-morbidity under the current Health Service Executive (HSE) guideline, one must recognise the many similarities these youth have with those that are, including poor nutritional status, potential cardiac compromise and decreased bone marrow function alongside persistent food restriction and high exercise expenditure. It is long recognised that youth with AN have lowered immunity. Cytopenias and bone marrow changes are commonly observed, the severity correlating with the degree of weight loss (Sabel *et al*. 2013). Patients often present with anaemia, leucopenia or thrombocytopenia in a pattern involving one, two or all three cell lines simultaneously (Hütter *et al*. 2009). Nutritionally compromised patients with AN may also have peripheral circulatory problems, or vasculitis, now being recognised as an atypical and possibly late presentation in COVID-19 (Nirenberg and del Mar Ruiz Herrera 2020).

Until more is known about the effect of COVID-19 in youth with AN, it is imperative that routine practice is altered to minimise unnecessary physical contact and hence potential exposure to the virus, yet ensure safe physical monitoring, and alertness to atypical clinical presentations. Clinicians should have a lower threshold for considering infectious complications because signs of infection such as fever may be absent, leading to delayed diagnosis and treatment. They should also enquire about new onset peripheral circulatory problems. Although weight restoration leads to normalisation of blood counts, this happens over weeks to months, placing them at high risk during the COVID-19 pandemic.

Therefore, at the onset of the Government imposed population-based restrictions (27th March), the SIMPLE model had to be adapted, and information sent to all families. Parents were advised that wherever possible, home-based intensive weekly FBT sessions by telephone or zoom would replace clinic/hospital attendances. Strict infection prevention and HSE control policies would be followed by the use of personal protective equipment whenever clinical attendance and physical contact was necessary.

Parental assistance was sought to support non-medical therapists make accurate clinical assessments remotely, using a structured clinical review developed by the authors (Table 2). This included twice weekly weighing, accurately recording food intake to allow daily calorie estimates to be calculated, estimate degree of energy expenditure and collecting vital signs when possible. Post-FBT review, clinicians used a traffic light system to grade clinical risk (red, orange or green) and discussed this with the FBT consultant/medical specialist to ensure that ongoing OPD non-face-to-face treatment was justified, despite potential clinical risk. Clinically indicated investigations or medical review was organised on a case-by-case basis, avoiding attendance at an otherwise busy emergency department. Adherence to general HSE advice when at home, such as practising social distancing and hand hygiene, was reinforced. As per standard care, parents are reminded of how to contact the clinic if they are concerned or advised to contact their GP or local emergency department if any urgent medical issues arise.

**Table 2.** SIMPLE structured parental monitoring of youth with AN^*^

Weekly cardiac (orthostatic symptoms) and respiratory (COVID-19 symptoms) review as well as assessment for any new physical symptoms (e.g. fatigue and dizziness)
Twice weekly weight checks using a digital scale first thing in the morning post voiding. This enabled accurate recording of their dry weight
Weekly heart rate checks using a fit bit or following clinician’s instructions or web-based suggestions (https://www.mayoclinic.org/healthy-lifestyle/fitness/expert-answers/heart-rate/faq-20057979) (https://www.childrens.com/health-wellness/is-your-childs-heart-rate-healthy)
Accurate recording of their daily calorie and fluid intake, to allow estimates of daily intake to be balanced with energy expenditure, and help inform therapists’ advice
Monitoring of compensatory behaviours, such as exercise, vomiting or laxative use and any changes to this, as they may adversely affect the physical state

* Devised by the authors.

By maintaining close contact and collaboration with parents, it was hoped to keep face-to-face visits and carer burden to a minimum whilst still providing the highest standard of clinical care and support possible.

Although the organisation has had electronic health records in place for many years, telemedicine had not traditionally been used. There were initial reservations in the HSE about data protection issues using Zoom and an initial reliance was placed on telephone consultations. To address any concerns with regard to data protection and staff confidence in using telepsychiatry, a SIMPLE post-telephone/video consultation checklist was devised, conforming with recently published Medical Protection Society (MPS) advice (2020) (Table 3). This reminded staff to establish and document the identity of the informant, describe telepsychiatry limitations, ensure appropriate time and setting for clinical encounters and confirm informed consent. MDT team members working from home were also able to document their method of safe and timely transfer of clinical data to the patient’s medical notes.

**Table 3.** The Medical Protection Society^*^

If you have doubts about your ability to adequately assess the patient remotely, or in cases of emergency, you must recommend the most appropriate route for them to seek medical assistance
You should document that you are satisfied that you can adequately assess them remotely in the clinical records
Remote consultations should preferably occur with patients already known to you, where you have full access to their clinical records
For new patients, you must request their medical records, and if unable to access them you must consider whether you can adequately assess them and document this
Both clinician and parents/patient must be able to reliably identify each other
The reason for remote consultation must be explained to the patient
It is your responsibility to ensure you practise in accordance with any applicable laws and regulations around the diagnosis, treatment, prescription and provision of therapy/medication to patients

* The Medical Protection Society. https://www.medicalprotection.org/ireland/resources/articles/view/covid-19-and-remote-consultations-how-we-can-help. 2020 Mar 16

The enforced restrictions on society in attempts to limit the spread of the pandemic have been severe. They include quarantine, social distancing practices, and closure of all but essential commercial outlets, school and university and recreational venues. The negative psychological effect of quarantine and social isolation has been well documented, with youth particularly at risk of symptoms of post-traumatic stress disorder (Brooks *et al*. 2020).

These adverse effects might be experienced most acutely by families who have mental health difficulties. Lack of routine, lack of social contact, infection fears and uncertainty around the illness and duration of restrictions may contribute to anxiety and depression in already vulnerable groups. In youth with AN, deterioration in mental health well-being may negatively impact on their motivation and ability to recover from their AN. Fears of low personal control, postulated by some to be an aetiological risk for AN, and applicable at this time, may trigger an increase in weight control behaviours in youth to compensate for this (Tiggemann & Raven, 1998). Heightened expressed emotions as a result of lockdown, or an inability to escape the watching eyes of parents at every meal, the inability to go outside and exercise, be distracted by school or social contact may all lead to a deterioration in youth well-being. Bodywhys, the national voluntary organisation supporting those affected by eating disorders, has reported an increase in eating disordered thoughts during the lockdown among their members (2020).

However, it is also possible that for some spending more time at home than with peers, absence of such intense social comparison during social contact, might in fact be welcome. Parents may also value the opportunity to be able to witness and supervise all meals, and having both parents at home, for some might facilitate an alignment in parental approaches. Anecdotal evidence from case reviews in CAMHS suggests that some youth with social anxiety and bullying at school have adapted very well to the school closures.

Caregiver burden in parents of youth with eating disorders is already recognised to be higher than in other psychiatric disorders (Graap et al., 2008) and enlisting their help for closer physical monitoring at this time may increase this further. Given the risk of adverse psychological effects and risk of decompensation during this pandemic, it is essential that healthcare providers adapt their services, such as outlined above, to maintain the safe and effective ongoing delivery of FBT and parental support. It is essential that careful consideration is given to balancing the protective effect of few or no clinical attendances, with the risk on unrecognised medical deterioration or excessive carer burden. Further research will be required to capture the impact of COVID-19 in this cohort and devise appropriate supports to prepare for further potential pandemics. Whilst informal feedback has been positive, the authors hope to establish the perspectives of families and providers in due course.
